# Naturalist's Monthly Report

**Published:** 1811-03

**Authors:** 


### NATURALIST'S MONTHLY REPORT.

### JANUARY.

### *Reviving Winter Month.*

——————The horizontal sun

Broad o'er the south, hangs at his utmost noon,

And, ineffectual, strikes the gelid cliff.

The wind was easterly from the 1st to the 10th of the present month. In the afternoon of the 10th it was south, and on the 11th westerly. On the 12th it was full south, and afterwards south-west; and from the 13th to the 23d for the most part westerly, or north-west. Towards the latter part of the 23d it changed to the north; and on the 24th and 25th was north-east. It was north-west on the 26th and 27th, and easterly during the last four days of the month.

We had strong gales on the 4th, 5th, 6th, 7th, 12th, 17th, and 30th, and fresh gales on the 1st, 8th, 13th, 14th, 15th, 23d, 24th, 25th, 27th, and 31st.

There was rain on the 10th, 12th, 13th, 14th, 15th, 17th, 18th, and 31st; and snow on the 1st, 2d, 3d, 28th, and 30th.

The weather was frosty on the 1st, 2d, 3d, 4th, 5th, 6th, 7th, 8th, 9th, 22d, 26th, 27th, 28th, and 29th, 30th; and hazy or foggy on the 10th, 12th, 14th, 17th, and 21st.

In the early part of the month I observed several bulfinches about the hedges, an occurrence which I have seldom remarked except in the extreme cold weather of winter.

January 3d. The starlings, which, in the beginning of November, were much more numerous than they usually are in this neighbourhood, continue apparently undiminished in numbers.

January 8th. Fieldfares are very numerous.

January 10th. Notwithstanding the long continuance of easterly gales and frost, the wild fowl which have come in are hitherto very few. Wild-geese have been exceedingly scarce. One wild swan (*anas cygnus ferus* of Linnæus) has been shot.

The frost has been so hard that the rivers are frozen. The frost broke up on the 10th.

January 15th. A great quantity of what is denominated, in this neighbourhood, ground ice, is now floating down both the Avon and Stour. This ice is formed at the bottom of the water, and is known by the roots and leaves of water plants which it carries along with it. Many persons have been much perplexed to account for the formation of this ice.

January 17th. The weather is now so warm that spiders come out of their hiding places and stand upon their webs; and the house flies have in some degree recovered from their torpidity.

January 19. I this day saw advertised in one of the London Papers, that a single dealer in wild-fowl had just received, for sale, 10,000 Bernacle nacle geese! The 4000 mallards, 204 cranes, 204 bitterns, 400 herons, 200 pheasants, 500 partridges, 400 woodcocks, and 100 curlews, which are stated to have been served up at Archbishop Nevill's famous intronization feast, in the year 1466, were, I think, scarcely more remarkable.

January 25. Snowdrops and primroses are in flower under the sunny walls of warm and sheltered gardens; and the flower-buds of the mezereon are nearly ready to burst.

January 27. The only salmon which has been caught during the present month, was taken on this day. It weighed twenty four pounds.

*Hampshire.*

